# Potentially Inappropriate Medication Use in Older Patients in Swiss Managed Care Plans: Prevalence, Determinants and Association with Hospitalization

**DOI:** 10.1371/journal.pone.0105425

**Published:** 2014-08-19

**Authors:** Oliver Reich, Thomas Rosemann, Roland Rapold, Eva Blozik, Oliver Senn

**Affiliations:** 1 Department of Health Sciences, Helsana Group, Zurich, Switzerland; 2 Institute of General Practice and Health Services Research, University Hospital, Zurich, Switzerland; 3 Department of Primary Medical Care, University Medical Center Hamburg-Eppendorf, Hamburg, Germany; University of Glasgow, United Kingdom

## Abstract

**Objectives:**

To describe the prevalence and determinants of potentially inappropriate medication (PIM) use and association with hospitalizations in an elderly managed care population in Switzerland.

**Methods:**

Using health care claims data of four health insurers for a sample of managed care patients 65 years of age and older to compare persons on PIM with persons not on PIM. Beers' 2012 and PRISCUS criteria were used to determine the potential inappropriateness of prescribed medications. The sample included 16′490 elderly patients on PIM and 33′178 patients not on PIM in the time period of January 1, 2008 through December 31, 2012. Prevalence estimates are standardized to the population of Switzerland. Associations between PIM and hospitalizations were examined by multivariate Cox regression analyses controlling for possible confounding variables.

**Results:**

The estimated prevalence of PIM use in our managed care sample was 22.5%. Logistic regression analysis showed that number of different medications used in the previous year, total costs in the previous year and hospitalization in the previous year all significantly increased the likelihood of receiving PIM. Multiple Cox regression analysis revealed that those on cumulative levels of PIM use acted significantly as a factor related to greater hospitalization rates: the adjusted HR was 1.13 (95% CI 1.07–1.19) for 1 PIM, 1.27 (95% CI 1.19–1.35) for 2 PIM, 1.35 (95% CI 1.22–1.50) for 3 PIM, and 1.63 (95% CI 1.40–1.90) for more than 3 PIM compared to no PIM use.

**Conclusions:**

The prevalence of PIM in managed care health plans are widely found but seem to be much lower than rates of non-managed care plans. Furthermore, our study revealed a significant association with adverse outcomes in terms of hospitalizations. These findings stress the need for further development of interventions to decrease drug-related problems and manage patients with multiple chronic conditions.

## Background

Prescribing potentially inappropriate medications (PIM) can lead to adverse drug events (ADE), significant morbidity and mortality, and may increase health care expenditures [1]–[5].

The elderly are at particular risk for inappropriate drug prescription. Many of the older persons suffer from chronic conditions that necessitate the use of multiple drugs [6]. In particular, the use of multiple medication increases the risk of prescribing PIM for elderly [7]–[9]. The physiologic changes in pharmacokinetics and pharmacodynamics in old age go together with polypharmacy and PIM and contribute to a higher risk of ADEs [10]. In Switzerland, people of 65 years of age or older account for 17.2% of the total population and it is estimated that this percentage will increase to 24% by 2030 [11]. Hence, the prevention and recognition of drug-related problems in the elderly represents an area of concern in the delivery of medical care and will be a principal challenge in clinical practice in the upcoming years.

A number of studies of the elderly in various settings have presented data indicating potentially inappropriate drug prescribing, with prevalences of up to 28% in community-dwelling elderly and up to 40% in nursing home residents, and have shown that a large proportion of hospital admissions and mortality are a result of ADEs [12]–[21]. A high prevalence of potentially inadequate medication (PIM) use in the community-dwelling older population in Switzerland has been recently reported [22]. Little is known about medication-related problems in a managed care setting. There are only a few studies, which used different approaches to estimate the prevalence or association of PIM on different outcomes in managed care populations in the U.S. [4], [23]–[25]. However, the prevalence and determinants of potentially inappropriate medication use and the impact of these on various adverse outcomes in the elderly managed care population have not been previously evaluated in Switzerland. In 2014 nearly 58% of the Swiss population were enrolled in managed care models illustrating the increasing significance of integrated health provision in Switzerland [26]. Social health insurance is compulsory for the population in Switzerland. Basic insurance allows the insured person the freedom of choice of doctors in the outpatient sector and unlimited access to physicians. Alternative forms of insurance exist with the option of restrained choice of medical providers granting policyholders discounts on the basic premium if they agree to sign up to managed care models and only consult certain providers. Reich et al. have described the main forms of managed care models found in Switzerland in detail [27].

This study used population-based claims data to evaluate prescribing for older adults in managed care health plans and defined potentially inappropriate medications using the updated 2012 Beers criteria [28] and the PRISCUS list [29]. The objectives of this study were to determine the prevalence and determinants of PIM use and the hospitalizations associated with incident PIM use in older patients in managed care health plans in Switzerland.

## Methods

### Study design, sample and data source

The database of this study included health care claims data from four statutory health and accident insurance companies in Switzerland (Helsana Group) during a 5-year study period, from January 1, 2008, until December 31, 2012. The study population was extracted from a dataset of circa 1.2 million persons (year 2012) with mandatory health insurance across Switzerland, which provides an approximate representativeness to the general population (proportion of 15 percent of 8.04 million inhabitants). All health care invoices submitted to Helsana for reimbursement were considered. Since the recorded insurance claims cover almost all health care invoices, these data are highly reliable. We estimate that 2–3% of all claims invoices are paid directly by the patient (e.g. due to high deductibles chosen) and not reimbursed by the health insurer. We conducted a retrospective claims data analysis, identifying 239,075 community-dwelling individuals age 65 and older (Switzerland: 1.31 million elderly). All elderly persons (49,668 individuals) enrolled in a managed care plan at individual incident PIM date during the study period were finally included in our study. We built our PIM definition on preliminary work performed [22] and used the updated Beers criteria [28] and the PRISCUS list [29] to identify and measure PIM use. Each drug prescription provided a unique identifier for the individual as an incident PIM date. In order to focus our analysis on incident PIM use, individuals who received any PIM prescription medication in the year before their individual incident PIM date were excluded from the sample.

### Statistical analysis

The prevalence rates of PIM use in the community-dwelling elderly population were calculated per age group. This was done for the Beers criteria and the PRISCUS list separately and in combination. All rates were adjusted for differences between the Helsana sample and the Swiss general population using census data from the Swiss Federal Office of Statistics [30]. In order to compare our rates in a managed care population with prior research findings (in the general population), we applied the PRISCUS list and actual Beers criteria as well as the old Beers definition to our prevalence rates [31]. Furthermore, the Beers criteria is the most widely used internationally and the PRISCUS list for the northern neighbor Germany to assess medication therapy adequacy in the elderly [20]. Other and newer geriatric PIM criteria have been devised and validated such as the STOPP/START criteria [32]. The STOPP/START criteria, however, requires clinical information on diagnosis (e.g. chronic obstructive pulmonary disease, glaucoma), which is unfortunately not available in our claims data and therefore cannot be applied in this study.

We calculated descriptive statistics to illustrate characteristics of the study population by comparing persons on PIM with persons not on PIM. Differences between the two groups were assessed by the two-sample Wilcoxon-test and chi-squared test. Concurrent PIM use was defined as number of PIM prescriptions within individual incidence year. To control for differences in health status among the sample we considered pharmacy based cost groups (PCG) [33]. PCGs are widely used to control for confounding by chronic diseases when clinical diagnosis information is lacking [34]–[36]. We entered the number of different chronic diseases per individual into the analyses as a numerical variable.

Logistic regression analysis was performed to identify the determinants for PIM exposure, where PIM usage (0 or 1) was the dependent variable and the independent variables included age, gender, deductible chosen, number of different drugs taken, number of chronic diseases, total costs in the previous year and acute hospital admission in the previous year.

In addition, for the analysis of the time until the occurrence of an adverse event (all-cause acute hospital admissions within 1 year of individual incident prescription in days after initial PIM prescription), we used hazard ratios (HR) as effect measures and their 95% confidence intervals (CIs), calculated by means of Cox proportional hazards regression models. We applied a multivariate Cox regression model to adjust for morbidity and other potential confounders. Possible interactions between covariates (e.g. number of concurrent PIM use*number of different medication previous year; number of concurrent PIM use*number of different chronic conditions) were assessed. Including these interactions resulted in increased hazard ratios for the main effects of PIM exposure. The interaction term was marginally below 1, accounting for a reduction of the effect of concurrent PIM in combination with the other interaction variable. This first increase in the estimates with subsequent reduction due to interactions are much more cumbersome to interpret with no effect on the conclusion. Therefore we decided to use the reported model without interaction variables. A two-sided p-value <0.05 was considered significant. All statistical analyses performed using R, version 2.14.2.

### Ethics

In compliance with the Swiss Federal Law on data protection, all data were anonymized and de-identified to protect the privacy of patients, physicians, and hospitals. Because the data were retrospective, pre-existing, and de-identified, this study was exempted from ethics committee approval.

## Results

**Figure 1:**
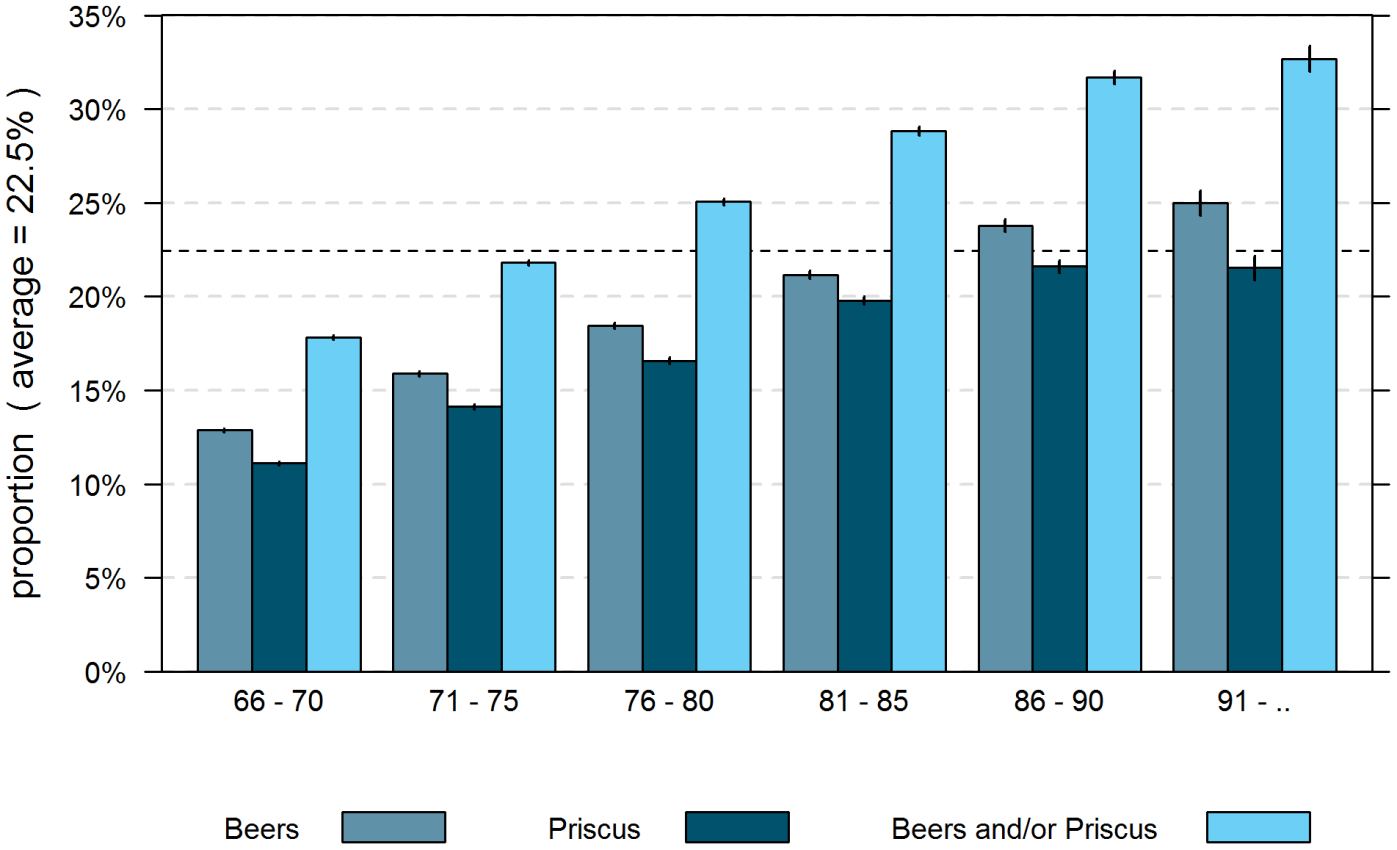
Proportion of persons in managed care models aged more than 65 years receiving PIM with 95 % confidence interval; years 2008-2012 (standardised for Swiss population); new Beers criteria.

**Figure 2:**
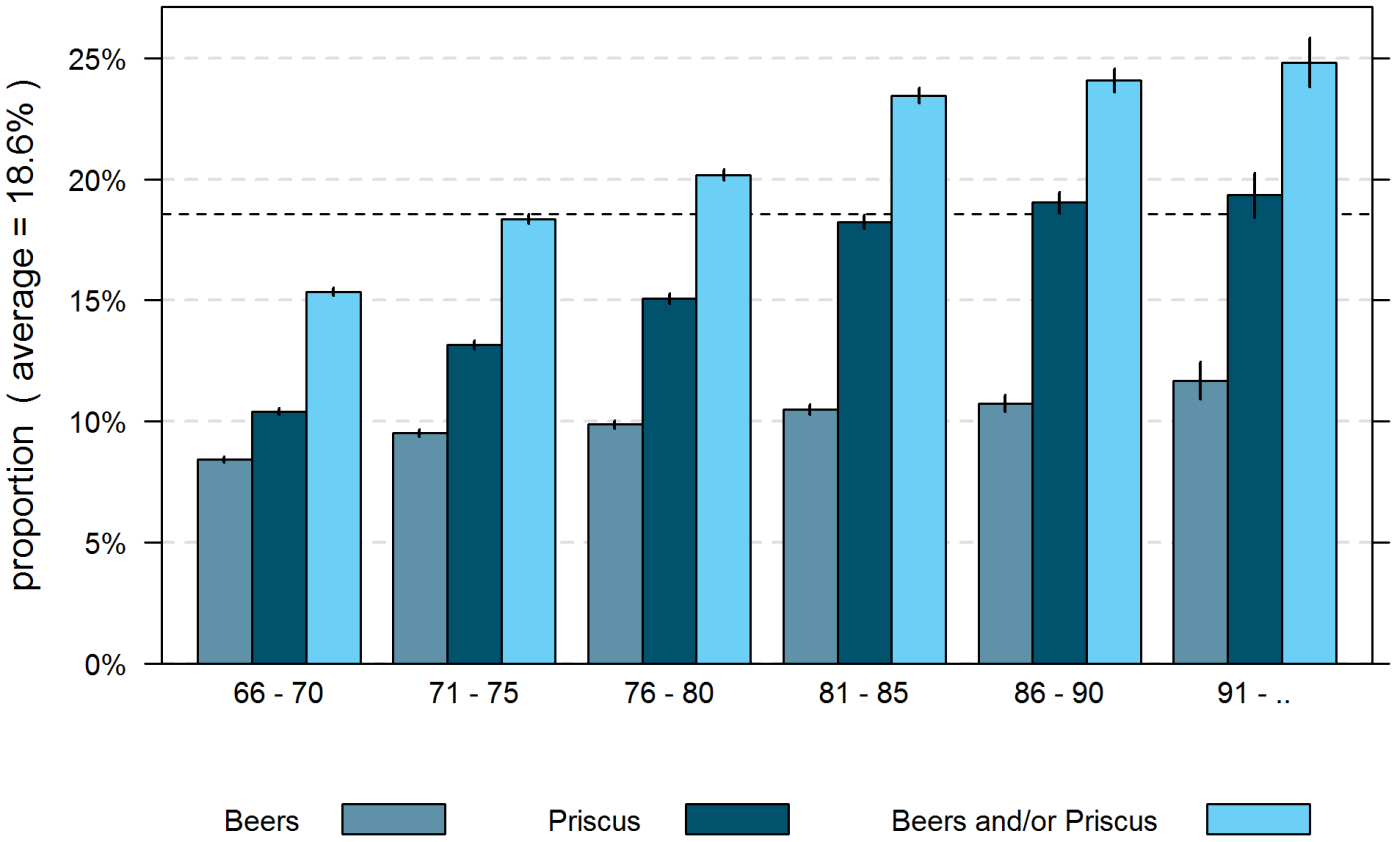
Proportion of persons in managed care models aged more than 65 years receiving PIM with 95% confidence interval; years 2008–2010 (standardised for Swiss population); old Beers criteria.

Based on reimbursement data and adjusted for differences between Helsana enrollees and the total population in Switzerland in terms of age, gender, and canton of residence, 22.5% of the community-dwelling population aged more than 65 years in managed care models received at least one medication which is potentially inappropriate according to the updated 2012 Beers criteria or PRISCUS list. Figure 1 shows the proportion of individuals per age group and gender who were prescribed a PIM. The effect of the revised Beers criteria on the prevalence of PIM is depicted in figure 2 showing a lower overall prevalence of PIM use of 18.6% in this standardized managed care plan population according to the 2003 Beers criteria.

Descriptive statistics for the incident PIM cases are displayed in table 1. The number of managed care insured who were newly prescribed a PIM during the study period was 16,490. Significant differences between those prescribed and those not prescribed a PIM were found for sex, age, deductible class, number of different medication used, number of chronic conditions, total costs previous year, and number of concurrent PIM. However, the variable hospitalization in the previous year was not found to be related to be different between the two groups (p = 0.787). During the study period, the observed overall incidence of adverse outcome as in hospitalizations was 21% for the whole study population. The rate for individuals in the PIM-group was 25.5% compared with 18.7% in the Non-PIM use group (p<0.001).

**Table 1:** Characteristics and hospitalizations in elderly Swiss managed care patients with potentially inappropriate medication (PIM) use versus non-PIM use, years 2008–2012.

	PIM use	Non-PIM use	Total	
Variables	(n = 16′490)	(n = 33′178)	(n = 49′668)	p-Value^a^
Gender [no. (%)]				<0.001
Female	7′798 (47.3%)	14′449 (43.5%)	22′247 (44.8%)	
Male	8′692 (52.7%	18′729 (56.5%)	27′421 (55.2%)	
Age [mean ±SD]	74.3 (6.3)	74.8 (6.6)	74.6 (6.5)	<0.001
Deductible [no. (%)]				<0.001
Low (CHF 300, 500)	14′120 (85.6%)	27′786 (83.7%)	41′906 (84.4%)	
High (>CHF 500)	2′370 (14.4%)	5′392 (16.3%)	7′762 (15.6%)	
No. of different medication used	11.8 (7.1)	9.2 (7.8)	10.1 (7.7)	<0.001
[mean ±SD]				
No. of chronic diseases	3.5 (1.8)	2.9 (2.2)	3.1 (2.1)	<0.001
[mean ±SD]				
Total costs previous year	5′736; 2′972 (9′162)	5′540; 2′673 (9′264)	5′605; 2′779 (9′231)	<0.001
[mean, median, ±SD]				
Hospitalization previous year	3′572 (21.7%)	5′687 (17.1%)	9′259 (18.6%)	0.787
[no. (%)]				
≤2 days	1′153 (7.0%)	1′786 (5.4%)	2′939 (5.9%)	
>2 days	2′772 (16.8%)	4′422 (13.3%)	7′194 (14.5%)	
No. of concurrent PIM (within incidence year)				<0.001
[no. (%)]				
0	0 (0%)	33′178 (100%)	33′178 (66.8%)	
1	11′004 (66.7%)	0 (0%)	11′004 (22.2%)	
2	3′964 (24.0%)	0 (0%)	3′964 (8.0%)	
3	1′109 (6.7%)	0 (0%)	1′109 (2.2%)	
>3	413 (2.5%)	0 (0%)	413 (0.8%)	
Hospitalizations within 1 year[no. (%)]	4′211 (25.5%)	6′216 (18.7%)	10′427 (21.0%)	<0.001

a Two-sample Wilcoxon-tests for age, no. of different medication used, no. of chronic diseases, total costs previous year; ×2-tests for comparing difference between the two groups PIM use and Non-PIM use.

Applying multivariate logistic regression analysis, the factors that exhibited significant associations with PIM prescriptions included the following (table 2): age 81–85 years (OR 0.86; 95% CI 0.78–0.94), age 86–90 years (OR 0.68; 95% CI 0.60–0.78), age 91+ years (OR 0.73; 95% CI 0.57–0.93), 1–4 different medication used previous year (OR 1.82; 95% CI 1.53–2.15), 5–10 different medication used previous year (OR 2.14; 95% CI 1.76–2.59), 11–20 different medication used previous year (OR 1.94; 95% CI 1.57–2.39), number of chronic diseases (OR 0.34–0.86), total costs previous year (OR 1.99–2.71) and hospitalization previous year (OR 1.5; 95% CI 1.35–1.67). However, the variables of gender and deductible class lost their significance in this analysis.

**Table 2:** Results of multivariate logistic regression analysis of determinants for PIM exposure in elderly managed care patients in Switzerland, years 2008–2012.

Independent variables	Odds ratio	95% CI	p-Value
Gender			
Male			
Female	0.95	0.90–1.01	0.092
Age			
…65–70 years	1.00 (reference)		
…71–75 years	0.95	0.88–1.02	0.180
…76–80 years	1.01	0.93–1.10	0.745
…81–85 years	0.86	0.78–0.94	0.002 **
…86–90 years	0.68	0.60–0.78	0.000 ***
…91+ years	0.73	0.57–0.93	0.012 *
Deductible			
Low (CHF 300, 500)	1.00 (reference)		
High (>CHF 500)	1.02	0.94–1.11	0.656
No. of different medication used previous year			
None	1.00 (reference)		
…1–4	1.82	1.53–2.15	0.000 ***
5–10	2.14	1.76–2.59	0.000 ***
11–20	1.94	1.57–2.39	0.000 ***
21+	1.04	0.81–1.33	0.778
No. of chronic diseases			
None	1.00 (reference)		
1	0.86	0.74–0.98	0.030 *
2	0.76	0.66–0.88	0.000 ***
3	0.71	0.61–0.83	0.000 ***
4–6	0.54	0.46–0.63	0.000 ***
7+	0.34	0.27–0.42	0.000 ***
Total costs previous year			
None	1.00 (reference)		
Group 1	1.99	1.73–2.28	0.000 ***
Group 2	2.54	2.18–2.95	0.000 ***
Group 3	2.36	2.02–2.76	0.000 ***
Group 4	2.71	2.31–3.19	0.000 ***
Group 5	2.57	2.18–3.03	0.000 ***
Group 6	2.59	2.17–3.09	0.000 ***
Group 7	2.24	1.86–2.71	0.000 ***
Group 8	2.31	1.87–2.85	0.000 ***
Group 9	2.30	1.81–2.92	0.000 ***
Hospitalization previous year			
No	1.00 (reference)		
Yes	1.50	1.35–1.67	0.000 ***

**Figure 3:**
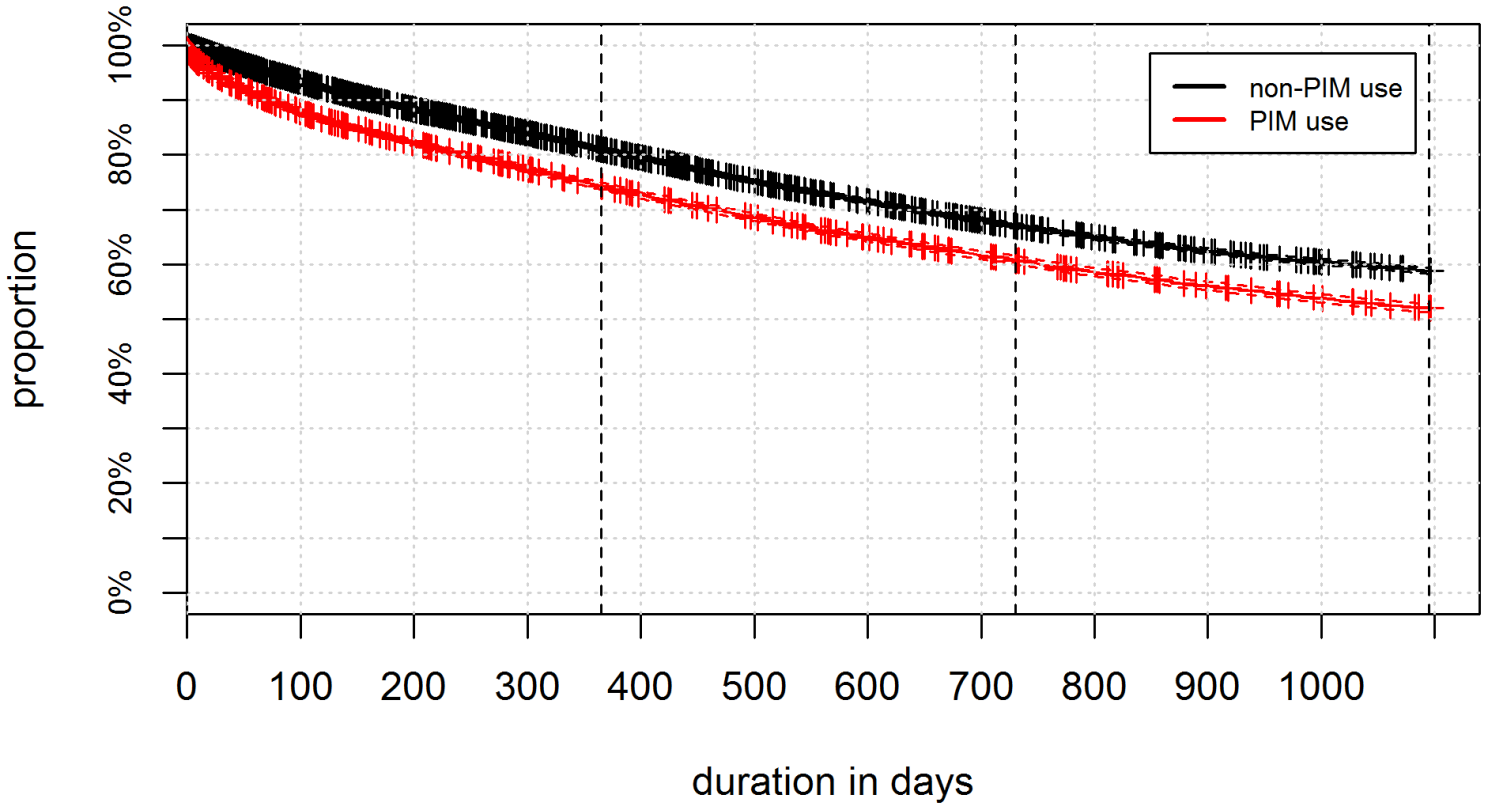
Kaplan-Meier curve comparing persons in managed care models aged more than 65 years receiving PIM and time to first hospitalization and persons without PIM-prescription and time to first hospitalization in Switzerland, years 2008–2012.

Table 3 presents the proportional hazard model for hospitalization within one year after incident PIM use. The analysis revealed that potentially inappropriate medication use was significantly associated with hospitalization. The adjusted hazard ratios (HR) for those on cumulative levels of PIM use were: 1.13 (95% CI 1.07–1.19) for 1 PIM, 1.27 (95% CI 1.19–1.35) for 2 PIM, 1.35 (95% CI 1.22–1.50) for 3 PIM, and 1.63 (95% CI 1.40–1.90) for more than 3 PIM compared to no PIM use. Increasing age, as well as polypharmacy and having high costs in the previous year increased the hazard ratio, whereas females and the variable high deductible class had a decreased HR for hospitalization. The variables number of chronic disease and hospitalization in the previous year had no significant association with hospitalization. In addition, figure 3 displays a Kaplan-Meier curve comparing persons receiving PIM and time to first hospitalization and persons without PIM-prescription and time to first hospitalization.

**Table 3:** Results of Cox regression analysis for determinants and adverse outcome hospitalization in elderly managed care patients in Switzerland, years 2008–2012.

	Hospitalization		
Independent variables	Hazard ratio	95% CI	p-Value
No of concurrent PIM use			
0	1.00 (reference)		
1	1.13	1.07–1.19	0.000 ***
2	1.27	1.19–1.35	0.000 ***
3	1.35	1.22–1.50	0.000 ***
>3	1.63	1.40–1.90	0.000 ***
Gender			
Male	1.00 (reference)		
Female	0.89	0.85–0.93	0.000 ***
Age			
…65–70 years	1.00 (reference)		
…71–75 years	1.09	1.03–1.16	0.004 **
…76–80 years	1.21	1.13–1.28	0.000 ***
…81–85 years	1.34	1.25–1.43	0.000 ***
…86–90 years	1.50	1.37–1.64	0.000 ***
…91+ years	1.68	1.44–1.96	0.000 ***
Deductible			
Low (CHF 300, 500)	1.00 (reference)		
High (>CHF 500)	0.91	0.85–0.98	0.013 *
No. of different medication used			
previous year			
None	1.00 (reference)		
…1–4	1.15	0.98–1.35	0.083
5–10	1.24	1.04–1.48	0.018 *
11–20	1.42	1.18–1.70	0.000 ***
21+	1.56	1.27–1.91	0.000 ***
No. of chronic diseases			
None	1.00 (reference)		
1	0.94	0.83–1.05	0.267
2	0.97	0.86–1.09	0.563
3	0.91	0.80–1.03	0.137
4–6	1.00	0.89–1.14	0.955
7+	1.10	0.94–1.29	0.225
Total costs previous year			
…None	1.00 (reference)		
Group 1	1.23	1.08–1.41	0.003 **
Group 2	1.40	1.22–1.61	0.000 ***
Group 3	1.43	1.24–1.65	0.000 ***
Group 4	1.43	1.24–1.66	0.000 ***
Group 5	1.56	1.35–1.81	0.000 ***
Group 6	1.75	1.50–2.03	0.000 ***
Group 7	1.83	1.56–2.13	0.000 ***
Group 8	1.88	1.60–2.22	0.000 ***
Group 9	1.88	1.57–2.25	0.000 ***
Hospitalization previous year			
No	1.00 (reference)		
Yes	1.01	0.94–1.08	0.778

## Discussion

Our study revealed a standardised PIM prevalence rate of 22.5% in elderly managed care patients according to the updated Beers criteria and PRISCUS list. This finding is in concordance with earlier studies with a large proportion of the non-managed care population with inappropriate medications [37]–[40]. For example, Blozik et al. [22] reported a high prevalence of PIM use of 21.1% for the community-dwelling elderly residents in Switzerland recently. The PIM criteria adopted in this mentioned study is however different to our research and the interpretation of the results have to be done with caution. Siebert et al. compared the application of selected PIM criteria in clinical routine and found the PRISCUS list was less sensitive than the application of STOPP criteria [41]. Bearing this in mind, our finding is therefore most probably underestimated and this suggests a much higher rate of prescription of PIM. The Swiss study of Blozik et al. used the 2003 Beers criteria to determine the overall prevalence rate, whereas our study is based on the updated 2012 Beers criteria. In the course of updating the Beers list, three additional medications have been included (glyburide, megestrol, and sliding-scale insulin) and, as the mentioned authors already assumed in their paper, this enhancement would lead to higher PIM prevalence rates. In order to be able to compare the two prevalence rates correctly, we applied the old Beers definition to our managed care sample and found a prevalence rate for PIM of 18.6%, which is significantly lower to the rate reported by Blozik et al.. It seems there does appear to be some greater risk awareness in prescribing potentially inappropriate medications in managed care plans compared to the general fee-for-service delivery models in Switzerland. Moreover, the significantly lower PIM prevalence rate might be due to the fact that managed care systems allow for improving care coordination. Previous research reveals the importance of care coordination [42] and indicates the great potential for continuous cost containment by applying managed care models [27]. However, prevalence rates from other study results (based on older or different PIM definitions and with much smaller patient samples) also examining persons in managed care plans in the United States are significantly higher than our rate (24.2–29%) [23]–[25], revealing the general difficulty of international comparisons in the different health care settings.

In our study, it appears that the probability of patients receiving potentially inappropriate medication is significantly associated with polypharmacy, total costs in the previous year and hospitalization in the previous year. Moreover, the multivariate analysis indicates that older persons and the persons with chronic diseases possess a lower probability for PIM exposure. Our study is supported by previous studies that have found that especially polypharmacy and female sex are important determinants for an increased likelihood of receiving a PIM prescription [38], [43]–[45]. In contrast to our study, however, Lin et al. [37] found a significant association between two further risk factors, advanced age and number of chronic diseases, and the likelihood of receiving inappropriate medications. An explanation for this may be the fact that their study was based on the inclusion of only elderly patients with a chronic disease who received long-term (3-month) prescriptions and using the Beers' 2002 criteria, thus, making it difficult to make a proper comparison. We suggest that within the Swiss managed care setting, physicians might be more cautious about avoiding PIM for older elders with chronic diseases and might put more emphasis on what to prescribe and on coordinating the respective medication for the treatment of the chronic diseases. Based on our findings and in line with previous research conducted [46]–[47], it seems to be important to reduce the number of medications prescribed in order to improve the safety of medication use in elderly managed care patients.

The last study objective was to examine the harmful impact of PIM exposure in the managed care population, since little evidence exists regarding adverse events such as hospitalizations. Therefore, all-cause acute hospital admissions within 1 year of individual incident prescription (in days after initial PIM prescription) was used as outcome, controlling for morbidity and other possible confounders. There was significant and increasing association between the number of concurrent PIM use and hospitalizations. Those on a PIM had significantly higher risk of a hospitalization than those not on an inappropriate medication. This result is consistent with findings from other studies [5], [37], [39], [48]–[52]. Several other factors such as advanced age, high number of different medications and total costs in the previous year were also found to be associated with more frequent hospitalizations. Additional research is necessary to prove causation and the results of this study cannot assess the appropriateness of drug prescribing. Nevertheless, focusing on the identification of PIM use can play an important role in risk management improvement efforts within the medical practice.

This study is one of few investigating the outcome of hospitalizations of PIM use in an older managed care population, and to our knowledge the only national study to provide a comprehensive overview about the situation of PIM prevalence in this specific health plan type in Switzerland. Although our data set is extremely broad and comprehensive, there are a number of limitations that influence the conclusions that can be drawn. First, our study was limited to a managed care population that may not be generalizable to other populations. Second, detailed clinical information on the patients were not available. However, drug-based diagnoses are a valid proxy for medical diagnoses and widely used in epidemiological and outcomes research to control for various chronic conditions. Third, using information from claims data restricted our ability to guarantee that the medications were genuinely taken by the individual patient and not dispensed in the garbage for example. Lastly, the information in our claims data may be slightly underrepresented as about 3% of the used claims invoices were paid directly by the patients and not by the health insurer.

In conclusion, the prevalence of PIM use is substantially lower in Swiss managed care populations compared to the general fee-for-service health delivery, which could signify better performance in drug prescription. The probability of harmful outcome as in hospitalizations occurring in patients with PIM exposure was much higher than in those not receiving PIM.

Our research suggests that managed health care plans in Switzerland offer a powerful model for improving medication prescriptions. These plans have found a way to engage the right set of physicians around the objective of delivering optimal and coordinated health care. Coordinated care helps ensure that patients, especially the chronically ill, get the right care at the right time, with the goal of avoiding unnecessary duplication of services and preventing adverse outcomes. Our research demonstrates that managed care plans potentially deliver more appropriate polypharmacy care than uncoordinated medicine and thus do a better job of improving health care value.
